# Real-world efficacy and prognostic factors of lenvatinib plus PD-1 inhibitors in 378 unresectable hepatocellular carcinoma patients

**DOI:** 10.1007/s12072-022-10480-y

**Published:** 2023-02-08

**Authors:** Xu Yang, Bowen Chen, Yanyu Wang, Yunchao Wang, Junyu Long, Nan Zhang, Jingnan Xue, Ziyu Xun, Linzhi Zhang, Jiamin Cheng, Jin Lei, Huishan Sun, Yiran Li, Jianzhen Lin, Fucun Xie, Dongxu Wang, Jie Pan, Ke Hu, Mei Guan, Li Huo, Jie Shi, Lingxiang Yu, Lin Zhou, Jinxue Zhou, Zhenhui Lu, Xiaobo Yang, Yilei Mao, Xinting Sang, Yinying Lu, Haitao Zhao

**Affiliations:** 1 Department of Liver Surgery, State Key Laboratory of Complex Severe and Rare Diseases, Peking Union Medical College Hospital, Chinese Academy of Medical Sciences and Peking Union Medical College (CAMS & PUMC), Beijing, 100730 China; 2 Peking University 302 Clinical Medical School, Beijing, China; 3 Comprehensive Liver Cancer Center, The Fifth Medical Center of the PLA General Hospital, Beijing, 100039 China; 4 Tianjin Medical University Cancer Institute and Hospital, Tianjin, China; 5 Guizhou Medical University, Guiyang, China; 6 Department of Radiology, Peking Union Medical College Hospital, Chinese Academy of Medical Sciences and Peking Union Medical College, Beijing, China; 7 Center of Radiotherapy, Peking Union Medical College Hospital, Chinese Academy of Medical Sciences and Peking Union Medical College, Beijing, China; 8 Departmentof Medical Oncology, Peking Union Medical College Hospital, Chinese Academy of Medical Sciences and Peking Union Medical College, Beijing, China; 9 Department of Nuclear Medicine, Peking Union Medical College Hospital, Chinese Academy of Medical Sciences and Peking Union Medical College, Beijing, China; 10 Department of Pathology, Peking Union Medical College Hospital, Chinese Academy of Medical Sciences and Peking Union Medical College, Beijing, China; 11 Senior Department of Oncology, The Fifth Medical Center of the PLA General Hospital, Beijing, China; 12 Department of Hepatobiliary and Pancreatic Surgery, The Affiliated Cancer Hospital of Zhengzhou University and Henan Cancer Hospital, Zhengzhou, China; 13 Hepatobiliary and Pancreatic Surgery, Shenzhen Qianhai Shekou Free Trade Zone Hospital, Shenzhen, China

**Keywords:** Hepatocellular carcinoma, Un-resectable, Lenvatinib, PD-1 inhibitor, Pembrolizumab, Nivolumab, Adverse events, Hepatitis B virus

## Abstract

**Introduction:**

Combining lenvatinib with a programmed cell death protein-1 (PD-1) inhibitor has been explored for the treatment of un-resectable hepatocellular carcinoma (uHCC). This study aimed to investigate the real-world efficacy of and prognostic factors for survival associated with lenvatinib plus PD-1 inhibitor treatment in a large cohort of Asian uHCC patients even the global LEAP-002 study failed to achieve the primary endpoints.

**Methods:**

Patients with uHCC treated with lenvatinib and PD-1 inhibitors were included. The primary endpoints were overall survival (OS) and progression-free survival (PFS), and the secondary endpoints were the objective response rate (ORR) and adverse events (AEs). Prognostic factors for survival were also analyzed.

**Results:**

A total of 378 uHCC patients from two medical centers in China were assessed retrospectively. The median patient age was 55 years, and 86.5% of patients were male. Hepatitis B virus (HBV) infection (89.9%) was the dominant etiology of uHCC. The median OS was 17.8 (95% confidence interval (CI) 14.0–21.6) months. The median PFS was 6.9 (95% CI 6.0–7.9) months. The best ORR and disease control rate (DCR) were 19.6% and 73.5%, respectively. In multivariate analysis, Child‒Pugh grade, Barcelona Clinic Liver Cancer stage, Eastern Cooperative Oncology Group performance status score, involved organs, tumor burden score, and combination with local therapy were independent prognostic factors for OS. A total of 100% and 57.9% of patients experienced all-grade and grade 3/4 treatment-emergent AEs, respectively.

**Conclusion:**

This real-world study of lenvatinib plus PD-1 inhibitor treatment demonstrated long survival and considerable ORRs and DCRs in uHCC patients in China. The tolerability of combination therapy was acceptable but must be monitored closely.

**Supplementary Information:**

The online version contains supplementary material available at 10.1007/s12072-022-10480-y.

## Introduction

Hepatocellular carcinoma (HCC) has a high incidence and mortality. Most cases are un-resectable HCC (uHCC) [1, 2]. Patients with uHCC treated with systematic therapy exhibit a median overall survival (OS) of only 11.8–21.2 months based on both phase III studies [3–10] and real-world studies [11–14].

Recently, phase 1b studies of lenvatinib plus a PD-1 inhibitor (pembrolizumab or nivolumab) for the treatment of uHCC patients showed promising efficacy in European and American [15] and Japanese cohorts [16]. Additionally, the recent LEAP-002 study found that compared with lenvatinib, lenvatinib plus pembrolizumab did not significantly increase OS (21.2 vs. 19.0 months, HR = 0.840, *p* = 0.0227 > 0.0185) but did result in the longest OS in patients with uHCC [10]. In East Asia, especially China, where chronic hepatitis B virus (HBV) infection is an important etiological factor of HCC and where the disease is different from that in other countries [1, 17], the efficacy of lenvatinib plus PD-1 inhibitor combination therapy is unclear.

Many PD-1 inhibitors for patients with uHCC are approved for use in China [18–20]. However, there is a lack of studies of large Chinese uHCC cohorts to evaluate this combination therapy. Moreover, it is unclear whether such patients could achieve better survival with lenvatinib plus PD-1 inhibitor combination therapy. Therefore, we designed this study to retrospectively observe the effect of lenvatinib plus PD-1 inhibitor combination therapy in a large uHCC cohort and explore the prognostic factors for survival associated with this treatment.

## Patients and methods

### Study design and patients

We retrospectively collected data on consecutive patients with uHCC treated with lenvatinib plus PD-1 inhibitors from October 2017 to November 2021 at 2 tertiary care hospitals (Peking Union Medical College Hospital (PUMCH) and the Fifth Medical Center of the People's Liberation Army General Hospital (PLAGH)).

Patients were eligible for this study if they met the following criteria: patients were pathologically confirmed or confirmed by imaging to have HCC [21–23]; patients exhibited at least one measurable lesion per the Response Evaluation Criteria in Solid Tumors (RECIST) version 1.1 guidelines; patients exhibited uHCC, i.e., were not eligible for curative treatment; patients were at least 18 years old; patients had a Child‒Pugh classification of A–B, and patients exhibited Eastern Cooperative Oncology Group (ECOG) performance status (PS) scores of 0–2. The exclusion criteria included the presence of end-stage HCC; history of organ transplant; prior lenvatinib or PD-1 inhibitor treatment; and discontinued use of combination therapy after less than 2 cycles of treatment. We performed a simple comparison with our real-world cohorts and similar randomized controlled LEAP-002 study to show the similarity and difference in baseline characteristics and clinical outcomes, which may also highlight some important clinical prognostic factors for survival.

This study is registered as NCT03892577.

### Treatment

Patients were treated with the de novo combination of lenvatinib and a PD-1 inhibitor. The dose of lenvatinib was dependent on patient weight (> = 60 kg: 12 mg; < 60 kg: 8 mg). For PD-1 inhibitors, pembrolizumab or nivolumab, and camrelizumab, sintilimab, toripalimab, or tislelizumab were allowed, and 200 mg (toripalimab: 240 mg), every three weeks, was administered intravenously. The choice of the type of PD-1 inhibitor in our study was a joint decision between physicians and patients in the real-world practice.

### Endpoints and assessments

The primary endpoints were OS and progression-free survival (PFS), and the secondary endpoints were the objective response rate (ORR) and safety. OS was defined as the time elapsed from the start of combination therapy until death (all causes). Surviving patients were censored at the last follow-up date. Tumor response was evaluated by the RECIST v1.1 guidelines [24]. PFS was defined as the time elapsed from the start of combination therapy until the date of progression or death (all causes), whichever occurred first. Durable clinical benefit (DCB) was defined as complete response (CR), partial response (PR), or stable disease (SD) for ≥ 24 weeks [25], which was evaluated by professional radiologists at our centers who were blinded to the therapeutic outcomes and clinicopathological features. Grades of adverse events (AEs) were assessed by physical examination and laboratory and imaging tests performed at the time of treatment based on the National Cancer Institute’s Common Toxicity Criteria (CTCAE) version 5.0. Management of AEs was according to the related guidelines [26, 27] and the guidelines for administration of the drug.

### Statistical analyses

Survival curves were estimated using the Kaplan‒Meier method and compared with the log-rank test. Univariate and multivariate Cox proportional hazard regression models were used to estimate the possible risk factors influencing PFS and OS; the results are reported as hazard ratios (HRs) and 95% confidence intervals (CIs). All variables potentially associated with OS or PFS and having a univariate p value of < 0.1 were included in multivariate analyses. The results with two-tailed p values of < 0.05 were considered statistically significant. Statistical analyses were performed using R version 4.1.2 and Statistical Package for the Social Sciences (version 25; IBM Corp., Armonk, NY).

## Results

### Patient characteristics

**Fig. 1:**
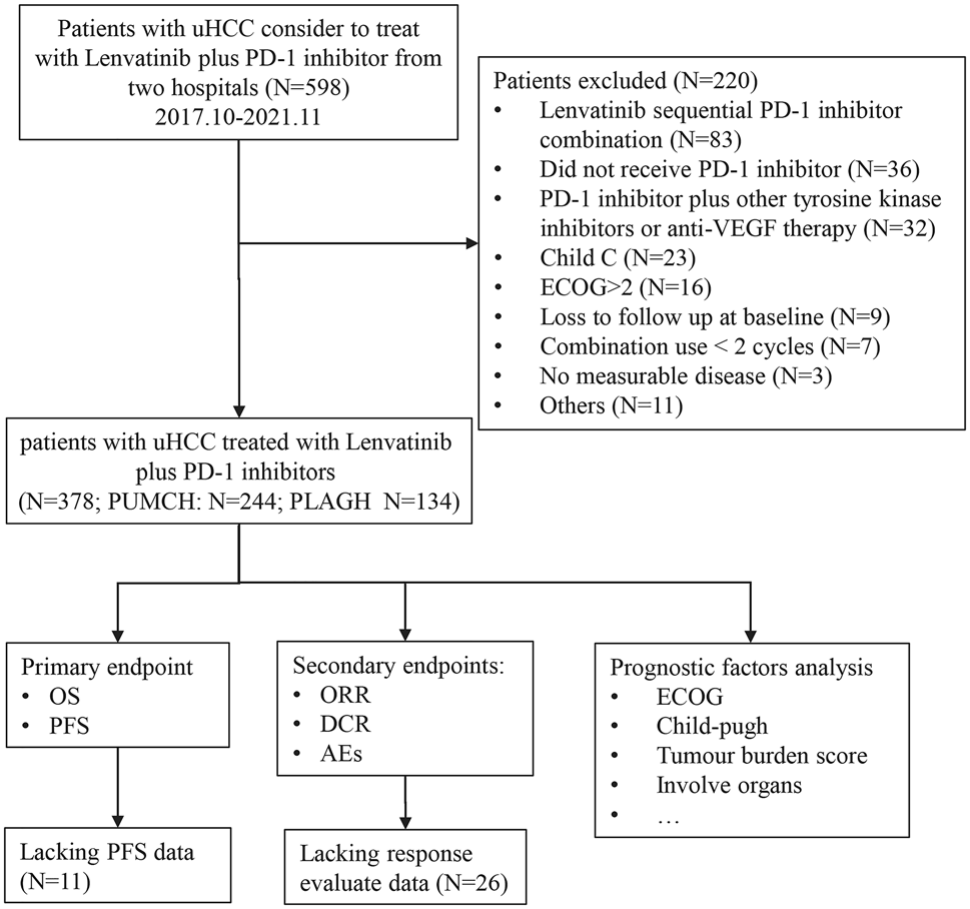
Flowchart of the study design

A total of 598 patients with HCC from October 2017 to November 2021 were screened from two hospitals, and 220 patients were excluded. Then, a total of 378 consecutively eligible uHCC patients who were treated with lenvatinib plus PD-1 inhibitors were evaluated (Fig. 1). Their baseline demographic and clinical characteristics are summarized in Table 1.

**Table 1:** Baseline demographic and clinical characteristics of Chinese un-resectable hepatocellular carcinoma (uHCC) patients and LEAP-002 study receiving lenvatinib plus PD-1 inhibitors

	Present study	LEAP-002 study	
Variable	Lenvatinib plus PD-1 inhibitors (*N* = 378)	Lenvatinib plus pembrolizumab (*N* = 395)	Lenvatinib plus placebo (*N* = 399)
Median age (range)	55 (18–89)	66 (19–88)	66 (20–88)
Sex—no. (%)			
Male	327 (86.5)	317 (80.3)	327 (82.0)
Female	51 (13.5)	78 (19.7)	72 (18.0)
ECOG performance status—no. (%)			
0	165 (43.7)	268 (67.8)	273 (68.4)
1	164 (43.4)	127 (32.2)	126 (31.6)
2	49 (13.0)	0 (0)	0 (0)
Child–Pugh Grade—no. (%)			
A	293 (77.5)	393 (99.5)	397 (99.5)
B	85 (22.5)	2 (0.5)	2 (0.5)
BCLC Stage—no. (%)			
B	48 (12.7)	85 (21.5)	95 (23.8)
C	330 (87.3)	310 (78.5)	302 (75.7)
Etiology—no. (%)			
HBV	340 (89.9)	192 (48.6)	193 (48.4)
HCV	11 (2.9)	94 (23.8)	87 (21.8)
HBV and HCV	1 (0.3)	0 (0)	0 (0)
Others	26 (6.9)	109 (27.6)	119 (29.8)
MVI—no. (%)			
Yes	198 (52.4)	71 (18.0)	62 (15.5)
No	180 (47.6)	324 (82.0)	337 (84.5)
EHS metastasis—no. (%)			
Yes	173 (45.8)	249 (63.0)	243 (60.9)
No	205 (54.2)	146 (37.0)	156 (39.1)
MVI or EHS—no. (%)	297 (78.6)	268 (67.8)	262 (65.7)
AFP level—no. (%)			
< 400 ng/mL	199 (52.6)	119 (30.1)	132 (33.1)
≥ 400 ng/mL	179 (47.8)	276 (69.9)	267 (66.9)
No. of involved organs—no. (%)			
1	217 (57.4)	–	–
2	123 (32.5)	–	–
≥ 3	38 (10.1)	–	–
Tumor burden score (TBS)—no. (%)			
< 8	199 (52.6)	–	–
≥ 8	179 (47.4)	–	–
Tumor largest size—no. (%)			
< 7 cm	195 (51.6)	–	–
≥ 7 cm	183 (48.4)	–	–
Number of prior systemic therapies—no. (%)			
0	310 (82.0)	395 (100.0)	399 (100.0)
1	60 (15.9)	0 (0.0)	0 (0.0)
≥ 2	8 (2.1)	0 (0.0)	0 (0.0)
Combined with local therapy—no. (%)			
Yes	206 (54.5)	0 (0.0)	0 (0.0)
No	172 (45.5)	395 (100.0)	399 (100.0)

*AFP* alpha-fetoprotein, *BCLC* Barcelona Clinic Liver Cancer, *ECOG* Eastern Cooperative Oncology Group, *EHS* extra-hepatic spread, *HBV* hepatitis B virus, *HCC* hepatocellular carcinoma, *HCV* chronic hepatitis C virus, *MVI* macrovascular invasion

The median age of the 378 patients was 55 years, and the majority (86.5%) of patients were male. The percentages of patients with ECOG-PS values of 0, 1 and 2 were 43.7%, 43.4% and 13.0%, respectively. Chronic HBV infection (89.9%) was the dominant etiology of uHCC. At baseline, 198 (52.4%) patients exhibited macrovascular invasion (MVI) by the tumor, whereas 173 (45.8%) exhibited extra-hepatic spread (EHS) of the tumor. The tumor burden score (TBS) was calculated by the maximum tumor size and number of tumors in the liver [28, 29]. Using the cutoff of 8 [28, 29], 47.4% of patients were classified as the high TBS score group. Most uHCC patients were systemic therapy-naïve (82.0%). During treatment, 54.5% of patients also received local therapy (trans-arterial chemoembolization (TACE), radiofrequency ablation (RFA) or radiation therapy (RT)) before and after two months of the combination therapy. There were many kinds of PD-1 inhibitors used for our cohort. The proportions of patients treated with pembrolizumab, nivolumab, sintilimab, camrelizumab, toripalimab, and tislelizumab were 18.3%, 5.6%, 33.9%, 27.5%, 11.6%, and 3.2%, respectively. We found that the important characteristics (ECOG, BCLC stage, etc.) were similar in lenvatinib plus different PD-1 inhibitors groups (Table S1). Only a relatively higher proportion of patients with Child‒Pugh B liver function (49/129, 38.3%) were observed in lenvatinib plus sintilimab subgroup.

### Efficacy outcomes and prognostic factors and subgroup analyses for survival

**Fig. 2:**
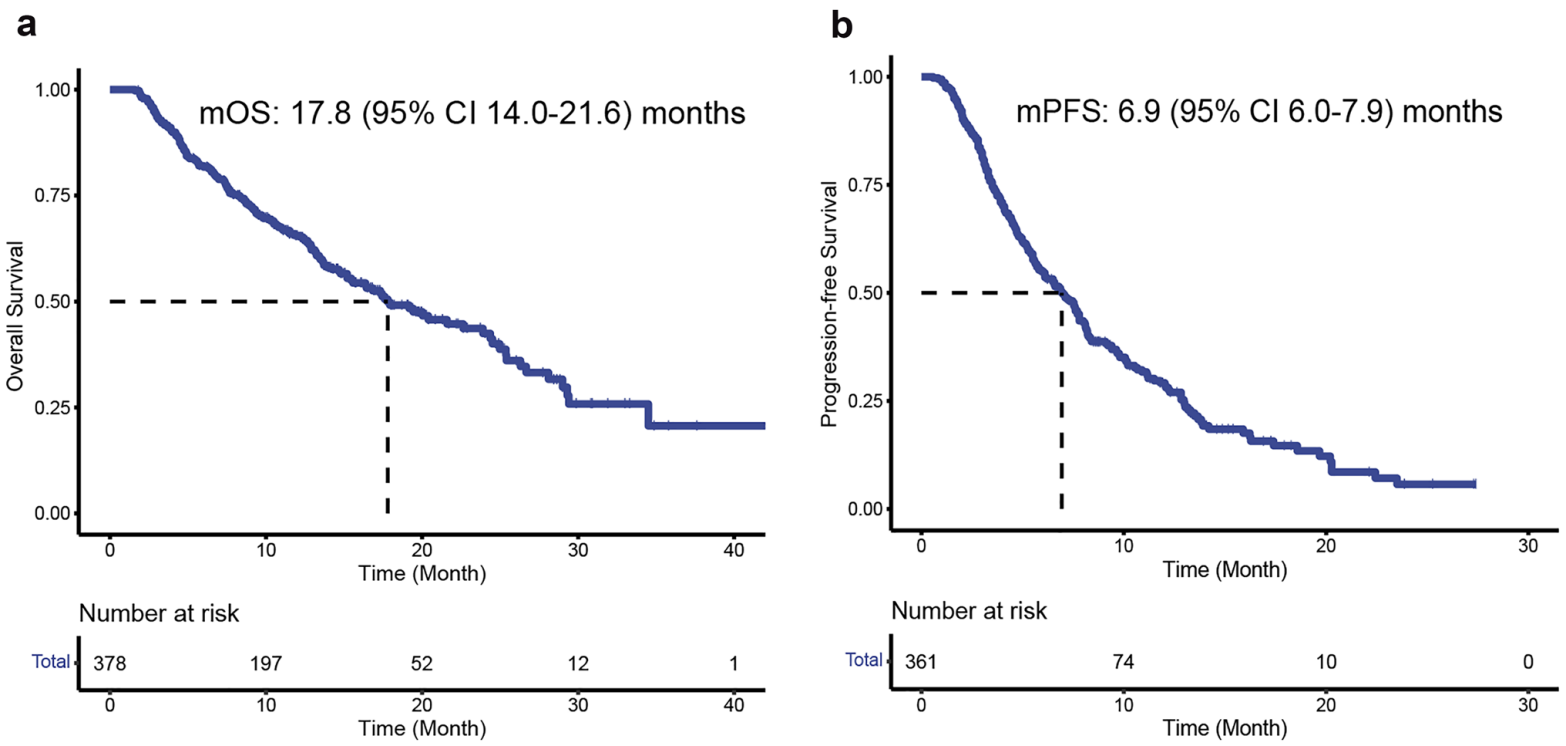
Kaplan‒Meier estimates of overall survival (**A**) and progression-free survival (**B**)

**Fig. 3:**
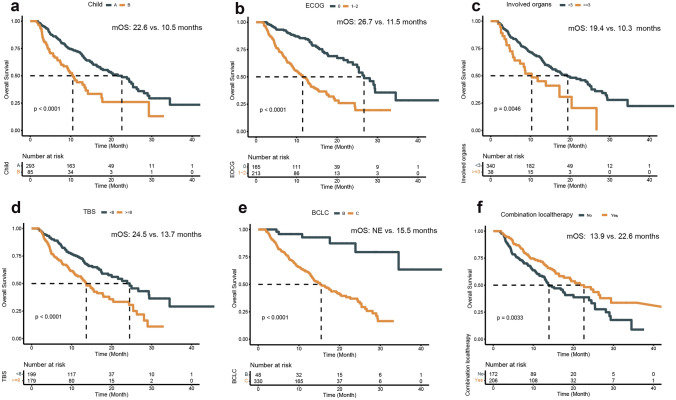
Kaplan‒Meier curves for overall survival stratified by Child‒Pugh classification (**A**), Eastern Cooperative Oncology Group (ECOG) performance status (PS) score (**B**), involved organs (**C**), tumor burden score (**D**), Barcelona Clinic Liver Cancer (BCLC) stage (**E**), and combination with local therapy (**F**) subgroups

At the time of analysis, the median follow-up was 10.4 (interquartile range (IQR) 6.2–15.8) months. The median OS was 17.8 months (95% confidence intervals (CIs) 14.0–21.6) (Fig. 2A), and the 1-year and 1.5-year OS rates were 43.7% (95% CI 38.7–48.7) and 18.3% (95% CI 14.4–22.1), respectively (Table 2). Eight potential prognostic variables for OS were selected based on univariate Cox analysis, namely Child‒Pugh grade, Barcelona Clinic Liver Cancer (BCLC) stage, ECOG PS, a-fetoprotein (AFP) level, involved organs, TBS, MVI, and combination with local therapy (Table 3). In multivariate analysis, Child‒Pugh grade (B vs. A: HR 1.675; 95% CI 1.171–2.396, *p* = 0.005; 10.5 vs. 22.6 months; Fig. 3A), ECOG PS (1–2 vs. 0: HR 2.209; 95% CI 1.538–3.173, *p* < 0.001; 11.5 vs. 26.7 months; Fig. 3B), involved organs (< 3 vs. ≥ 3: HR 1.716; 95% CI 1.073–2.744, *p* = 0.024; 10.3 vs. 19.4 months; Fig. 3C), and TBS (high vs. low: HR 1.543; 95% CI 1.093–2.177, *p* = 0.014; 13.7 vs. 24.5 months; Fig. 3D) were independently associated with a significantly shorter OS. Conversely, BCLC stage (B vs. C: HR 0.297; 95% CI 0.115–0.767, *p* = 0.012; not evaluated (NE) vs. 15.5 months; Fig. 3E) and combination with local therapy (yes vs. no: HR 0.665; 95% CI 0.485–0.911, *p* = 0.011; 22.6 vs. 13.9 months; Fig. 3F) were associated with a significantly longer OS (Table 3).

**Table 2:** Efficacy outcomes in Chinese un-resectable hepatocellular carcinoma (uHCC) patients and LEAP-002 study receiving lenvatinib plus PD-1 inhibitors

Parameter	Present study	LEAP-002 study	
	Lenvatinib plus PD-1 inhibitors (*N* = 378)	Lenvatinib plus pembrolizumab (*N* = 395)	Lenvatinib plus placebo (*N* = 399)
ORR, % (95 CI)	19.6 (15.6–23.6)	26.1%	17.5%
Best overall response			
CR, no. (%)	0 (0)	–	–
PR, no. (%)	74 (19.6)	–	–
SD, no. (%)	221 (58.5)	–	–
PD, no. (%)	57 (15.1)	–	–
Unknown/not evaluable, no. (%)	26 (6.9)	–	–
DCR, % (95 CI)	78.0 (73.9–82.2)	81.3%	78.4%
DCB, % (95 CI)	50.0 (45.0–55.0)	–	–
DOR, % (95 CI)	10.8 (7.5–14.0)	16.6 (range: 2.0 +–33.6 +)	10.4 (range: 1.9–35.1 +)
Median PFS, months (95%CI)	6.9 (6.0–7.9)	8.2 (6.4–8.4)	8.0 (6.3–8.2)
6 months, % (95 CI)	44.0 (38.9–49.2)	–	–
12 months, % (95 CI)	15.0 (11.3–18.6)	34.1%	29.3%
Median OS, months, months (95%CI)	17.8 (14.0–21.6)	21.2 (19.0–23.6)	19.0 (17.2–21.7)
6 months, % (95 CI)	75.4 (71.1–79.7)	–	–
12 months, % (95 CI)	43.7 (38.7–48.7)	–	–
18 months, % (95 CI)	18.3 (14.4–22.1)	–	–
Median follow-up, month (IQR)	10.4 (6.2–15.8)	32.1 (range: 25.8–41.1)	

*CI* confidence interval, *CR* complete response, *DCR* disease control rate, *DCB* durable clinical benefit, *DOR* duration of response, *IQR* interquartile range, *ORR* objective response rate, *OS* overall survival, *PD* progressive disease, *PFS* progression-free survival, *PR* partial response, *SD* stable disease

**Table 3:** Univariate and multivariate analyses of prognostic factors for progression-free survival (PFS) and overall survival (OS)

Variates	Univariate analysis for PFS	Multivariate analysis		Univariate analysis for OS	Multivariate analysis	
	*p* value	*p* value	HR (95% CI)	*p* value	*p* value	HR (95% CI)
Age (< 65 vs. ≥ 65)	0.116			0.552		
Sex (Female vs. Male)	0.979			0.710		
HBV (No vs. Yes)	0.793			0.854		
HCV (No vs. Yes)	0.454			0.829		
Child–Pugh score (B vs. A)	**0.050**	0.565	1.100 (0.795–1.523)	** < 0.001**	**0.005**	1.675 (1.171–2.396)
BCLC stage (B vs. C)	**0.001**	0.544	0.859 (0.525–1.404)	** < 0.001**	**0.012**	0.297 (0.115–0.767)
ECOG PS (1–2 vs. 0)	** < 0.001**	** < 0.001**	1.832 (1.363–2.461)	** < 0.001**	** < 0.001**	2.209 (1.538–3.173)
AFP level (≥ 400 vs. < 400)	**0.059**	0.488	1.098 (0.843–1.431)	**0.014**	0.474	1.122 (0.819–1.536)
Involve organs (≥ 3 vs. < 3)	0.113			**0.005**	**0.024**	1.716 (1.073–2.744)
TBS (≥ 8 vs. < 8)	**0.001**	**0.047**	1.348 (1.005–1.809)	** < 0.001**	**0.014**	1.543 (1.093–2.177)
MVI (Yes vs. No)	**0.004**	0.239	1.203 (0.885–1.636)	**0.001**	0.431	1.162 (0.800–1.689)
EHS (Yes vs. No)	0.367			0.153		
First line (No vs. Yes)	0.495			0.848		
Combination with local therapy (Yes vs. No)	**0.005**	**0.008**	0.701 (0.539–0.912)	**0.004**	**0.011**	0.665 (0.485–0.911)
PD-1 inhibitor (Others vs. Pembrolizumab)	0.451			0.332		

Bold values indicate *p* ≤ 0.05

*AFP* alpha-fetoprotein, *BCLC* Barcelona Clinic Liver Cancer, *CI* confidence interval, *ECOG* Eastern Cooperative Oncology Group, *EHS* extra-hepatic spread, *HBV* hepatitis B virus, *HCC* hepatocellular carcinoma, *HCV* chronic hepatitis C virus, *HR* hazard radio, *MVI* macrovascular invasion, *OS* overall survival, *PFS* progression-free survival, *TBS* tumor burden score

For PFS analysis, 361 patients were analyzed. The median PFS was 6.9 months (95% CI 6.0–7.9) (Fig. 2B), and the 0.5-year and 1-year PFS rates were 44.0% (95% CI 38.9–49.2) and 15.0% (95% CI 11.3–18.6), respectively. Based on multivariate analysis, ECOG PS (1–2 vs. 0: HR 1.832; 95% CI 1.363–2.461, *p* < 0.001; 5.1 vs. 10.1 months; Fig. S1A) and TBS (high vs. low: HR 1.348; 95% CI 1.005–1.809, *p* = 0.047; 5.4 vs. 8.2 months, *p* = 0.001; Fig. S1B) were associated with a significantly shorter PFS (Table 3). However, combination with local therapy (yes vs. no: HR 0.701; 95% CI 0.539–0.912, *p* = 0.008; 7.8 vs. 5.5 months; Fig. S1C) was an independent predictor of a longer PFS.

In the intent-to-treat analysis of 378 patients based on the RECIST v1.1 criteria, objective responses were observed in 74 patients (19.6%, 95% CI 15.6–23.6), and disease control was observed in 295 patients (78.0%, 95% CI 73.9–82.2). If the tumor exhibited a response, the median duration of response (DOR) was 10.8 (95% CI 7.5–14.0) months. Half (50%) of the patients reached the DCB from lenvatinib plus PD-1 inhibitor therapy.

### Safety

All patients were assessed for drug safety. The overall incidence of treatment-emergent adverse events (TEAEs) was 100% (Table 4). However, TEAEs were grade 3/4 in 219 (57.9%) patients. The most frequent grade 3 to 4 TEAEs (> 5%) were hypertension (15.1%), increased blood bilirubin levels (8.5%), fatigue (7.7%), proteinuria (7.1%), decreased platelet count (6.9%), decreased appetite (6.3%), hypokalemia (6.3%), and diarrhea (5.8%). Grade 5 fatal AEs occurred in 5 patients (1.3%) and included upper gastrointestinal bleeding (four patients) and cerebral hemorrhage (one patient). Generally, almost (99.7%, 377/378) all-grade AEs may refer to lenvatinib, and just 21.4% (81/378) of all-grade AEs may relate to PD-1 inhibitors. On the other hand, also almost (96.8%, 212/219) of grade 3 to 4 AEs may refer to lenvatinib and just 23.3% (51/219) of grade 3 to 4 AEs may relate to PD-1 inhibitors. Moreover, in our study, about 24.9% (94/378) of patients experienced treatment discontinued due to AEs. In addition, 19.6% of 378 patients were treated with systematic corticosteroids to manage AEs.

**Table 4:** Most common treatment-emergent adverse events in 378 Chinese un-resectable hepatocellular carcinoma (uHCC) patients receiving lenvatinib plus PD-1 inhibitors

Adverse events, *n* (%)	Any grade	Grade 3–4	Grade 5
Treatment-emergent adverse events	378 (100.0)	219 (57.9)	5 (1.3)*
Hypertension	185 (48.9)	57 (15.1)	
Increased blood bilirubin	162 (42.9)	32 (8.5)	
Fatigue	241 (63.7)	29 (7.7)	
Proteinuria	89 (23.5)	27 (7.1)	
Decreased platelet count	139 (36.8)	26 (6.9)	
Decreased appetite	299 (79.1)	24 (6.3)	
Hypokalemia	89 (23.5)	24 (6.3)	
Diarrhea	87 (23)	22 (5.8)	
Elevated aspartate aminotransferase	154 (40.7)	18 (4.8)	
Upper gastrointestinal bleeding	52 (13.8)	18 (4.8)	4 (1.1)
Hyponatremia	97 (25.7)	12 (3.2)	
Decreased leukocytes	99 (26.2)	11 (2.9)	
Rash	202 (53.4)	10 (2.6)	
Elevated alanine aminotransferase	160(42.3)	8 (2.1)	
Decreased weight	86 (22.8)	8 (2.1)	
Palmar-plantar erythrodysesthesia	60 (15.9)	7 (1.9)	
Pneumonia	19 (5.0)	7 (1.9)	
Hypoalbuminemia	198 (52.4)	6 (1.6)	
Pain	68 (18)	4 (1.1)	
Nausea	51 (13.5)	3 (0.8)	
Vomiting	40 (10.6)	2 (0.5)	
Dysphonia	30 (7.9)	2 (0.5)	
Pruritus	23 (6.1)	2 (0.5)	
Hypothyroidism	126 (33.3)	1 (0.3)	
Abdominal pain	82 (21.7)	1 (0.3)	
Fever	65 (17.2)	1 (0.3)	
Edema limbs	33 (8.7)	1 (0.3)	
Oral mucositis	32 (8.5)	1 (0.3)	
Periodontal disease	30 (7.9)	1 (0.3)	
Constipation	25 (6.6)	1 (0.3)	
Abdominal distension	49 (13.0)	0 (0.0)	
Epistaxis	13 (3.4)	0 (0.0)	

*Including cerebral hemorrhage (*N* = 1)

To clearly demonstrate AEs associated with lenvatinib plus different PD-1 inhibitors groups, we split AEs according to different treatment combinations (Table S2). The grade 3–4 TEAEs in lenvatinib plus pembrolizumab or nivolumab, sintilimab, camrelizumab, toripalimab, or tislelizumab were 56.5%, 81.0%, 57.0%, 57.7%, 56.8% and 41.7%, respectively, which is basically similar. For special ones, lenvatinib plus sintilimab group seems to have higher all-grade hypokalemia, hyponatremia and rash. Lenvatinib plus pembrolizumab seems to have higher all-grade hypokalemia and upper gastrointestinal bleeding. For lenvatinib plus camrelizumab group, the incidence of all-grade diarrhea may be higher and the incidence of reactive cutaneous capillary endothelial hyperplasia (RCCEP) as special AE for camrelizumab occurred in about 14.4% (15/104) patients.

## Discussion

To our knowledge, this is the largest real-world study of the use of lenvatinib plus PD-1 inhibitors in uHCC patients. We found that the median OS was 17.8 months and the median PFS was 6.9 months. The ORR and DCR were 19.6% and 73.5%, respectively. We also found that Child‒Pugh grade, BCLC stage, ECOG, involved organs, TBS, and combination with local therapy were independent prognostic factors for OS.

Many other cohort studies have also reported the efficacy of lenvatinib plus PD-1 inhibitors in uHCC patients. The phase I Keynote-524 study, the most representative study, reported that an ORR of 36.0% was reached in 100 uHCC patients treated with lenvatinib plus the PD-1 inhibitor pembrolizumab. Moreover, the median PFS and median OS were 8.6 months and 22.0 months, respectively [15]. However, the phage 3 LEAP-002 study found that compared with lenvatinib plus placebo in patients with uHCC, lenvatinib plus pembrolizumab did not significantly increase OS (21.2 vs. 19.0 months, HR 0.840, *p* = 0.0227 > 0.0185) [10]. The negative LEAP-002 study found that OS in the lenvatinib plus placebo arm (19.0 months) was longer than that in the lenvatinib arm (13.6 months) in the 2018 REFLECT study [4] due to higher rates (22.8%) and efficacy of sequential immunotherapy [10]. In our cohorts, 18.3% (69/378) of patients were treated with the same drug of lenvatinib plus pembrolizumab combination therapy as in the LEAP-002 study [10], but we did not find significant differences for lenvatinib plus other kinds of PD-1 inhibitor (*p* = 0.33) in our study. For lenvatinib plus sintilimab or camrelizumab, which is the most employed anti-PD-1 inhibitors in our study, some small cohorts found that the mPFS of this combination therapy is approximately 8.0–11.3 months [30–32], which is comparable with that reported in the mPFS in the LEAP-002 study (8.2 months) and our present study (6.9 months).

In the Keynote-524 study [15] and LEAP-002 study [10], patients were excluded if they had with Child–Pugh class B or C liver function, invasion at the main portal vein (Vp4), ECOG‒PS with 2 scores, or received prior systemic therapy. However, in present real-world cohort, 22.5% patients were with Child–Pugh class B, and 13.0% patients were with ECOG‒PS scores of 2, and 18.0% received prior systemic therapy. The efficacy of the combination therapy in our real-world cohort was lower than that achieved in the Keynote-524 study [15] and LEAP-002 [10] because we think important baseline characteristics (Child‒Pugh score, BCLC stage, ECOG PS scores, MVI) were better in these two studies than in our present study. However, such parameters may also be more realistic in real-world practice in Asian uHCC patients who have a high rate of HBV infection. We hope to get more details to compare our cohort with the LEAP-002 study when the LEAP-002 study was published "in extenso".

In clinical practice, the main concern is selecting patients who would benefit from the therapy [33]. We found that worse ECOG PS (1–2 vs. 0) was a negative prognostic factor for OS (HR = 2.209, *p* < 0.001) and PFS (HR = 1.832, *p* < 0.001). Patients with worse Child‒Pugh grades (B vs. A) had a shorter OS (HR = 1.675, *p* = 0.005) but not PFS (*p* > 0.05) in multivariate analysis. Many studies have found that the ECOG score and Child‒Pugh grade are prognostic factors for patients with uHCC who were administered lenvatinib and/or PD-1 inhibitors [34–36]. Wu et al. found in multivariate analyses that the Child‒Pugh class (Class B vs. A, HR = 2.646, *p* = 0.039) but not an ECOG score of ≥ 1 (HR = 1.889, *p* = 0.162) was a poor prognostic factor for survival in uHCC patients treated with lenvatinib plus pembrolizumab [35]. Choi et al. studied 203 Korean patients with uHCC treated with nivolumab and found that the Child‒Pugh B group had a shorter mOS (2.8 vs. 10.7 months; HR = 2.10; *p* < 0.001) but not mPFS (HR = 1.17, *p* = 0.430) [37]. Patients with worse ECOG PS or worse liver function might benefit less from lenvatinib plus PD-1 inhibitors, so the application of drugs should be done with caution.

Tumor characteristics are very important for survival in patients with uHCC [38]. We found that the involved organs and TBS may influence PFS and OS. In a post-analysis of the REFLECT study of patients with uHCC treated with lenvatinib or sorafenib, the number of tumor sites at baseline was a very important prognostic factor (*p* < 0.001) for OS in multivariate analysis [39]. Moreover, we found that combination loco-regional therapy was an independent factor for both better PFS and OS. This result was consistent with the results of previous studies that found that adding loco-regional therapy to a lenvatinib plus PD-1 inhibitor or lenvatinib monotherapy regimen could lead to a high response and long survival [9, 40–44].

The most frequent AEs were consistent with the use of lenvatinib monotherapy [4]. We think these common AEs may be related to the anti-vascular endothelial growth factor (VEGF) target mechanism [4, 45]. Regarding safety, ≥ grade 3 TEAEs need to be closely monitored. In the Keynote-524 study, grade 3 TEAEs were hypertension (18%), increased AST levels (14%), increased lipase levels (11%), diarrhea (7%), increased blood bilirubin levels (6% at level 3 and 2% at level 4), fatigue (6%), asthenia (6%), increased ALT levels (6%), decreased weight (5%) and proteinuria (5%) [15]. In the LEAP-002 study, 96.5% and 61.5% of uHCC patients underwent all-grade treatment-related adverse events (TRAE) and grade 3–4 TRAEs [10], respectively, which is similar to our study. However, in our study, 24.9% of treatment discontinuation due to AEs may be higher than about 18.0% in the Keynote-524 study [15] and LEAP-002 study [10]. It may be related to follow-up closely and real-world setting-based practice. We think careful management and adjustment of the drug dose may be important to address AEs and may prolong the duration of treatment and survival [46]. Notably in our cohort, fatigue, decreased appetite, and gastrointestinal bleeding may need closer monitoring and good management. Meanwhile, fatigue and decreased appetite may lead to low quality of life, while gastrointestinal bleeding is always life-threatening, especially in patients with chronic liver disease [47]. In real-world practice, doctors should be reminded to carefully monitor patients’ safety due to patients’ irregular visits and the influence of the coronavirus disease 19 (COVID-19) pandemic. There are several limitations in our study. First, potential bias could not easily be avoided due to the nature of the retrospective design. Second, multiple kinds of PD-1 inhibitors were heterogeneous and some were off-label used in the study; however, we did not find a significant difference when comparing the use of other PD-1 inhibitors with the use of pembrolizumab. Third, our cohort was predominantly HBV-infected uHCC patients, and the applicability of these findings to non-HBV-infected uHCC patients remains to be further validated in real-world practice.

## Conclusions

In conclusion, a real-world study found that lenvatinib plus PD-1 inhibitors achieved long survival and considerable response in uHCC patients in China. The tolerability of combination therapy was acceptable but should be monitored closely in real-world practice.

## Supplementary Information

Below is the link to the electronic supplementary material.

Supplementary file1 Figure S1. Kaplan‒Meier curves for progression-free survival stratified by Eastern Cooperative Oncology Group (ECOG) performance status (PS) score (A), tumor burden score (B), and combination with local therapy (C) subgroups. (PDF 836 KB)

Supplementary file2 (DOCX 26 KB)

Supplementary file3 (DOCX 27 KB)

## Data Availability

All data supporting the findings of this study are available in this article and its online supplementary material files. Further inquiries can be directed to the corresponding author.

## Abbreviations

AFP: Alpha-fetoprotein
ALT: Elevated alanine aminotransferase
AST: Aspartate aminotransferase
BCLC: Barcelona Clinic Liver Cancer
CI: Confidence interval
CR: Complete response
CTCAE: Common Terminology Criteria for Adverse Events
DCR: Disease control rate
DCB: Durable clinical benefit
DOR: Duration of response
ECOG: Eastern Cooperative Oncology Group
EHS: Extrahepatic spread
HBV: Hepatitis B virus
HCC: Hepatocellular carcinoma
HCV: Chronic hepatitis C virus
HAIC: Hepatic arterial infusion chemotherapy
IQR: Interquartile range
MVI: Macrovascular invasion
ORR: Objective response rate
OS: Overall survival
PD: Progressive disease
PFS: Progression-free survival
PD-1: Programmed death 1
PR: Partial response
RECIST: Response Evaluation Criteria in Solid Tumors
PLAGH: People’s Liberation Army General Hospital
PS: Performance status
PUMCH: Peking Union Medical College Hospital
SD: Stable disease
TBS: Tumor burden score
TACE: Transarterial chemoembolization
TEAE: Treatment-emergent adverse event
VEGF: Vascular endothelial growth factor
