# Supplementary figures and images for: Real-world efficacy and prognostic factors of lenvatinib plus PD-1 inhibitors in 378 unresectable hepatocellular carcinoma patients
Source: Hepatol Int. 2023 Feb 8;17(3):709–19. doi: 10.1007/s12072-022-10480-y (PMC9907200; doi:10.1007/s12072-022-10480-y)

# Figure S1

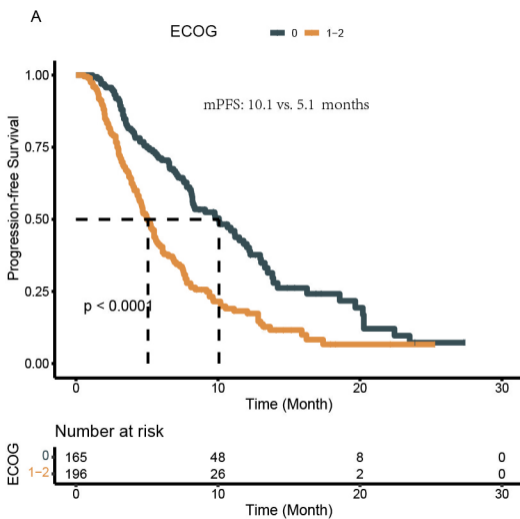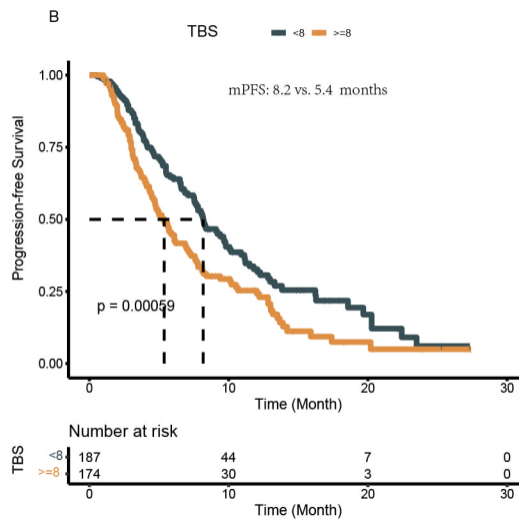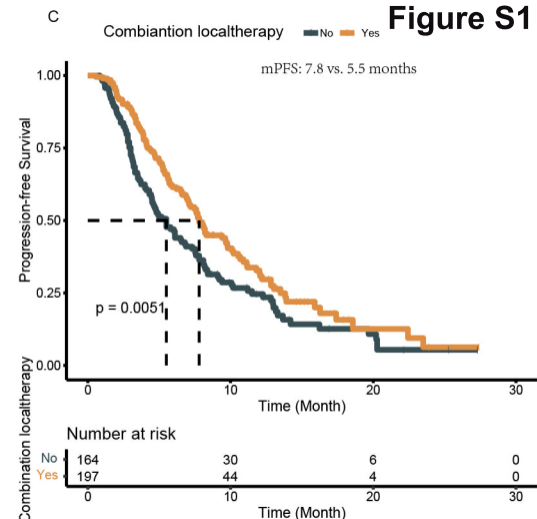

Supplement: Supplementary file 1 — Supplementary file1 Figure S1. Kaplan‒Meier curves for progression-free survival stratified by Eastern Cooperative Oncology Group (ECOG) performance status (PS) score (A), tumor burden score (B), and combination with local therapy (C) subgroups. (PDF 836 KB) [file 12072_2022_10480_MOESM1_ESM.pdf]
