# Supplementary material for: Real-world efficacy and prognostic factors of lenvatinib plus PD-1 inhibitors in 378 unresectable hepatocellular carcinoma patients
Source: Hepatol Int. 2023 Feb 8;17(3):709–19. doi: 10.1007/s12072-022-10480-y (PMC9907200; doi:10.1007/s12072-022-10480-y)
Supplement: Supplementary file 2 — Supplementary file2 (DOCX 26 KB) [file 12072_2022_10480_MOESM2_ESM.docx]

**Table S1.** Key baseline clinical characteristics of Chinese unresectable hepatocellular carcinoma (uHCC) patients treated with lenvatinib plus different PD-1 inhibitors

| **Variable** | **Pembrolizumab**  **(N=69)** | **Sintilimab**  **(N=128)** | **Camrelizumab (N=104)** | **Toripalimab**  **(N=44)** | **Nivolumab/ Tislelizumab (N=33)** | ***P* value*** | **All**  **(N=378)** |
| --- | --- | --- | --- | --- | --- | --- | --- |
| **ECOG performance status** — no. (%) |  |  |  |  |  | 0.525 |  |
| 0 | 36 (52.2) | 52 (40.6) | 46 (44.2) | 19 (43.2) | 12 (36.4) |  | 165 (43.7) |
| 1-2 | 33 (47.8) | 76 (59.4) | 58 (55.8) | 25 (56.8) | 21 (63.6) |  | 213 (56.3) |
| **Child-Pugh Grade** — no. (%) |  |  |  |  |  | **<0.001** |  |
| A | 56 (81.2) | 79 (61.7) | 87 (83.7) | 40 (90.9) | 31 (93.9) |  | 293 (77.5) |
| B | 13 (18.8) | 49 (38.3) | 17 (16.3) | 4 (9.1) | 2 (6.1) |  | 85 (22.5) |
| **BCLC Stage** — no. (%) |  |  |  |  |  | 0.537 |  |
| B | 5 (7.2) | 17 (13.3) | 14 (13.5) | 6 (13.6) | 6 (18.2) |  | 48 (12.7) |
| C | 64 (92.8) | 111 (86.7) | 90 (86.5) | 38 (86.4) | 27 (81.8) |  | 330 (87.3) |
| **No. of** **involved organs** — no. (%) |  |  |  |  |  | 0.818 |  |
| <3 | 64 (92.8) | 113 (88.3) | 95 (91.3) | 39 (88.6) | 29 (87.9) |  | 340 (89.9) |
| ≥3 | 5 (7.2) | 15 (11.7) | 9 (8.7) | 5 (11.4) | 4 (12.1) |  | 38 (10.1) |
| **Tumor burden score (TBS)** — no. (%) |  |  |  |  |  | 0.267 |  |
| <8 | 31 (44.9) | 65 (50.8) | 63 (60.6) | 21 (47.7) | 19 (57.6) |  | 199 (52.6) |
| ≥8 | 38 (55.1) | 63 (49.2) | 41 (39.4) | 23 (52.3) | 14 (42.4) |  | 179 (47.4) |
| **Combined with local therapy** — no. (%) |  |  |  |  |  | 0.881 |  |
| Yes | 37 (53.6) | 66 (51.6) | 61 (58.7) | 24 (54.5) | 18 (54.5) |  | 206 (54.5) |
| No | 32 (46.4) | 62 (48.4) | 43 (41.3) | 20 (45.5) | 15 (45.5) |  | 172 (45.5) |

*: Fisher’s exact test;

Abbreviations: BCLC, Barcelona Clinic Liver Cancer; ECOG, Eastern Cooperative Oncology Group; HCC, hepatocellular carcinoma
