# Supplementary material for: Real-world efficacy and prognostic factors of lenvatinib plus PD-1 inhibitors in 378 unresectable hepatocellular carcinoma patients
Source: Hepatol Int. 2023 Feb 8;17(3):709–19. doi: 10.1007/s12072-022-10480-y (PMC9907200; doi:10.1007/s12072-022-10480-y)
Supplement: Supplementary file 3 — Supplementary file3 (DOCX 27 KB) [file 12072_2022_10480_MOESM3_ESM.docx]

**Table S2.** Common treatment-emergent adverse events in unresectable hepatocellular carcinoma (uHCC) patients treated with lenvatinib plus different PD-1 inhibitors

|  | **Pembrolizumab (N=69)** | | **Nivolumab**  **(N=21)** | | **Sintilimab**  **(N=128)** | | **Camrelizumab (N=104)** | | **Toripalimab**  **(N=44)** | | **Tislelizumab**  **(N=12)** | | **All**  **(N=378)** | |
| --- | --- | --- | --- | --- | --- | --- | --- | --- | --- | --- | --- | --- | --- | --- |
| **Grade** | **Any grade** | **Grade 3-4** | **Any grade** | **Grade 3-4** | **Any grade** | **Grade 3-4** | **Any grade** | **Grade 3-4** | **Any grade** | **Grade 3-4** | **Any grade** | **Grade 3-4** | **Any grade** | **Grade 3-4** |
| **Treatment-emergent adverse events (%)*** | 100.0% | 56.5% | 100.0% | 81.0% | 100.0% | 57.0% | 100.0% | 57.7% | 100.0% | 56.8% | 100.0% | 41.7% | 100% | 57.9% |
| Hypertension | 58.0% | 15.9% | 33.3% | 14.3% | 53.1% | 12.5% | 43.3% | 17.3% | 50.0% | 18.2% | 25.0% | 8.3% | 48.9% | 15.1% |
| Increased blood bilirubin | 47.8% | 11.6% | 52.4% | 9.5% | 40.6% | 8.6% | 40.4% | 6.7% | 43.2% | 9.1% | 41.7% | 0.0% | 42.9% | 8.5% |
| Fatigue | 68.1% | 7.2% | 57.1% | 4.8% | 61.7% | 3.9% | 63.5% | 9.6% | 70.5% | 15.9% | 50.0% | 8.3% | 63.7% | 7.7% |
| Proteinuria | 27.5% | 4.3% | 28.6% | 14.3% | 33.6% | 11.7% | 13.5% | 2.9% | 15.9% | 6.8% | 0.0% | 0.0% | 23.5% | 7.1% |
| Decreased platelet count | 34.8% | 8.7% | 42.9% | 9.5% | 39.8% | 7.0% | 36.5% | 7.7% | 34.1% | 2.3% | 16.7% | 0.0% | 36.8% | 6.9% |
| Decreased appetite | 81.2% | 5.8% | 85.7% | 0.0% | 71.9% | 3.9% | 82.7% | 7.7% | 86.4% | 15.9% | 75.0% | 0.0% | 79.1% | 6.3% |
| Hypokalemia | 27.5% | 10.1% | 14.3% | 9.5% | 35.2% | 7.0% | 14.4% | 4.8% | 15.9% | 2.3% | 0.0% | 0.0% | 23.5% | 6.3% |
| Diarrhea | 17.4% | 7.2% | 23.8% | 14.3% | 14.8% | 2.3% | 31.7% | 8.7% | 29.5% | 4.5% | 41.7% | 0.0% | 23.0% | 5.8% |
| Elevated aspartate aminotransferase | 43.5% | 7.2% | 61.9% | 4.8% | 33.6% | 6.3% | 39.4% | 2.9% | 50.0% | 2.3% | 41.7% | 0.0% | 40.7% | 4.8% |
| Upper gastrointestinal bleeding | 23.2% | 4.3% | 33.3% | 19.0% | 7.8% | 3.1% | 9.6% | 3.8% | 15.9% | 4.5% | 16.7% | 8.3% | 13.8% | 4.8% |
| Hyponatremia | 23.2% | 7.2% | 28.6% | 0.0% | 36.7% | 4.7% | 20.2% | 1.0% | 13.6% | 0.0% | 8.3% | 0.0% | 25.7% | 3.2% |
| Decreased leukocytes | 33.3% | 7.2% | 38.1% | 9.5% | 21.9% | 0.8% | 25.0% | 2.9% | 25.0% | 0.0% | 25.0% | 0.0% | 26.2% | 2.9% |
| Rash | 52.2% | 5.8% | 23.8% | 4.8% | 78.1% | 0.8% | 46.2% | 1.0% | 20.5% | 4.5% | 33.3% | 8.3% | 53.4% | 2.6% |
| Elevated alanine aminotransferase | 43.5% | 1.4% | 66.7% | 0.0% | 30.5% | 3.9% | 43.3% | 1.9% | 59.1% | 0.0% | 50.0% | 0.0% | 42.3% | 2.1% |
| Decreased weight | 21.7% | 2.9% | 23.8% | 4.8% | 23.4% | 0.8% | 23.1% | 1.9% | 27.3% | 4.5% | 0.0% | 0.0% | 22.8% | 2.1% |
| Palmar-plantar erythrodysaesthesia | 23.2% | 2.9% | 14.3% | 0.0% | 13.3% | 2.3% | 12.5% | 1.0% | 22.7% | 2.3% | 8.3% | 0.0% | 15.9% | 1.9% |
| Pneumonia | 8.7% | 4.3% | 4.8% | 0.0% | 4.7% | 3.1% | 1.9% | 0.0% | 9.1% | 0.0% | 0.0% | 0.0% | 5.0% | 1.9% |
| Hypoalbuminemia | 58.0% | 2.9% | 57.1% | 0.0% | 51.6% | 3.1% | 54.8% | 0.0% | 45.5% | 0.0% | 25.0% | 0.0% | 52.4% | 1.6% |
| Pain | 17.4% | 0.0% | 28.6% | 0.0% | 13.3% | 2.3% | 17.3% | 1.0% | 22.7% | 0.0% | 41.7% | 0.0% | 18% | 1.1% |
| Nausea | 8.7% | 0.0% | 9.5% | 0.0% | 16.4% | 1.6% | 16.3% | 1.0% | 11.4% | 0.0% | 0.0% | 0.0% | 13.5% | 0.8% |
| Vomiting | 8.7% | 0.0% | 14.3% | 0.0% | 6.3% | 0.0% | 14.4% | 1.9% | 13.6% | 0.0% | 16.7% | 0.0% | 10.6% | 0.5% |
| Dysphonia | 13.0% | 0.0% | 0.0% | 0.0% | 4.7% | 0.8% | 5.8% | 1.0% | 20.5% | 0.0% | 0.0% | 0.0% | 7.9% | 0.5% |
| Pruritus | 8.7% | 0.0% | 0.0% | 0.0% | 7.0% | 0.0% | 4.8% | 0.0% | 6.8% | 4.5% | 0.0% | 0.0% | 6.1% | 0.5% |
| Hypothyroidism | 40.6% | 0.0% | 47.6% | 0.0% | 35.2% | 0.0% | 26.9% | 1.0% | 29.5% | 0.0% | 16.7% | 0.0% | 33.3% | 0.3% |
| Abdominal pain | 24.6% | 0.0% | 0.0% | 0.0% | 21.9% | 0.8% | 19.2% | 0.0% | 29.5% | 0.0% | 33.3% | 0.0% | 21.7% | 0.3% |
| Fever | 11.6% | 0.0% | 23.8% | 4.8% | 16.4% | 0.0% | 17.3% | 0.0% | 22.7% | 0.0% | 25.0% | 0.0% | 17.2% | 0.3% |
| Edema limbs | 8.7% | 0.0% | 19.0% | 0.0% | 6.3% | 0.0% | 8.7% | 1.0% | 11.4% | 0.0% | 8.3% | 0.0% | 8.7% | 0.3% |
| Oral mucositis | 8.7% | 0.0% | 4.8% | 0.0% | 9.4% | 0.0% | 7.7% | 0.0% | 11.4% | 2.3% | 0.0% | 0.0% | 8.5% | 0.3% |
| Periodontal disease | 8.7% | 0.0% | 0.0% | 0.0% | 4.7% | 0.0% | 8.7% | 0.0% | 15.9% | 0.0% | 16.7% | 8.3% | 7.9% | 0.3% |
| Constipation | 5.8% | 0.0% | 4.8% | 0.0% | 2.3% | 0.8% | 7.7% | 0.0% | 18.2% | 0.0% | 8.3% | 0.0% | 6.6% | 0.3% |
| Abdominal distension | 13.0% | 0.0% | 9.5% | 0.0% | 16.4% | 0.0% | 9.6% | 0.0% | 15.9% | 0.0% | 0.0% | 0.0% | 13% | 0.0% |
| Epistaxis | 1.4% | 0.0% | 4.8% | 0.0% | 2.3% | 0.0% | 6.7% | 0.0% | 2.3% | 0.0% | 0.0% | 0.0% | 3.4% | 0.0% |
| RCCEP | 0.0% | 0.0% | 0.0% | 0.0% | 0.0% | 0.0% | 14.4% | 0.0% | 0.0% | 0.0% | 0.0% | 0.0% | 4.0% | 0.0% |

*and including five grade 5 Treatment-emergent adverse events in which 1 cerebral hemorrhage for lenvatinib plus toripalimab, 4 upper gastrointestinal bleeding for lenvatinib plus pembrolizumab (N=1), sintilimab (N=2), toripalimab (N=1).

Abbreviation: RECCP: Reactive Cutaneous Capillary Endothelial Proliferation.
